# Exploring the Key Antecedents Influencing Consumer’s Continuance Intention toward Bike-Sharing Services: Focus on China

**DOI:** 10.3390/ijerph17124556

**Published:** 2020-06-24

**Authors:** Byoungsoo Kim, Daekil Kim

**Affiliations:** 1 School of Business, Yeungnam University, 280 Daehakro, Gyeongsansi 38541, Korea; kbsyu@yu.ac.kr; 2 School of Communications and Media, Seoul Women’s University, 621 Hwarangro, Nowon-Gu, Seoul 01797, Korea

**Keywords:** bike-sharing services, privacy risk, financial risk, sharing economy, perceived value

## Abstract

This study investigates the key antecedents affecting consumers’ continuance intention toward bike-sharing services in China. The theoretical framework clarifies the role of perceived value and trust in a service provider in enhancing customer’s continuance intention toward bike-sharing services. Perceived usefulness, perceived ease of use, and perceived enjoyment are considered vital factors in forming perceived value and trust in a service provider. Financial risk and privacy risk serve as inhibitors to consumers’ continuance intention. Our research model is validated using data from 224 bike-sharing consumers in China. Both perceived value and trust in a service have a significant impact on consumers’ continuance intention. However, financial risk significantly affects customer’s continuance intention, although privacy risk does not have a significant impact on it. The analysis results show that perceived usefulness has no significant effect on both perceived value and trust in a service provider. The results demonstrate that perceived ease of use and perceived enjoyment play a significant role in enhancing both perceived value and trust in a service provider. Our results are expected to provide academic and practical implications for bike-sharing services.

## 1. Introduction

Bike-sharing services are services offered for shared use to multiple consumers without them owning the bicycles [1,2]. Bike-sharing is considered as an alternative and convenient transport service offered to customers, which helps mitigate environmental problems such as energy depletion and climate change. From a consumer perspective, instead of purchasing bicycles, they can save money by conveniently renting bicycles from anywhere [3]. Because of these advantages, bike-sharing services operate in more than 400 cities around the world and are particularly popular in China [4,5]. Chinese service providers have targeted college students as their primary customers and started offering services on university campuses. Hellobike, Mobike, and Ofo are some of the major shared bicycle service providers in China. In 2015, the concept of shared bicycle service was introduced, commencing from tier 1 cities such as Beijing, Shanghai, and Shenzhen and expanding to tier 2 cities such as Qingdao, Chengdu, Nanjing, and Hefei [6]. The number of registered consumers of bike-sharing services increased from 1.8 billion in 2016 to 2.2 billion in 2017 and further to 2.35 billion in 2018 [6,7,8]. Initially, several bike-sharing service providers existed. However, after market consolidation through mergers and acquisitions, Hello, Mobike, and Ofo emerged as the largest players, together accounting for around 80% of the total market. Because of the intense market environment, it is important to understand the formation mechanisms of consumer’s continuance intention [9,10,11]. In this vein, this study investigates the key antecedents affecting consumers’ continuance intention toward bike-sharing services in China.

Perceived value and trust in a service provider are vital factors for the intention of continuous use in bike-sharing services. Perceived value is a key factor in using services that require spending on borrowing bikes [3,4,12]. Consumers are required to pay a deposit in advance when using the shared bicycle service in addition to paying for the usage time. Customers will ascribe a high level of value if there are many benefits derived from paying for the service [4,12]. Consumers’ trust in a service provider is expected to significantly impact their continuance intentions of using the services. In general, the low level of trust in shared bicycle service providers may result from a limited number of bicycles made available or negligent bicycle maintenance [4,9]. In particular, trust in a service provider is associated with a variety of activities, such as the condition of the bicycles and the function of mobile applications. In this context, perceived value and trust in a service provider are expected to play an important role in the formation of consumers’ continuance intention toward bike-sharing services.

This study posits perceived usefulness, perceived ease of use, and perceived enjoyment as leading factors affecting perceived values and trust in a service provider. Perceived usefulness and perceived enjoyment serve as external and internal motivations, respectively, when deciding whether to use the information system [5,11,12]. The unified theory of acceptance and use of technology model also reveals that perceived usefulness and enjoyment play a key role in the formation of an intention to accept and continue using information systems under various settings [12,13,14]. In the context of bike-sharing services, perceived usefulness and perceived enjoyment are expected to significantly shape perceived values and trust in a service provider. Ma et al. [5] showed that perceived ease of use is a factor considered in the technology acceptance model. They found that perceived ease-of-use and perceived usefulness have significant positive effects on customer’s attitudes toward bike sharing. Bike-sharing service providers undertake various efforts to provide service convenience, such as enabling consumers to unlock a bicycle and make payment through their mobile applications. This ease of use would positively influence perceived value and trust in a service provider [5,15].

Financial and privacy risk while using bike-sharing services serve as deterrents to continued use [3,11]. The financial risk emerges from the obligation of a user to pay a deposit in advance to use the bike-sharing service without expecting a refund upon service termination. In particular, with the announcement in 2018 and 2019 of bleak prospects for bike-sharing services in China, many consumers demanded a refund of the deposit. Therefore, we examine the effects of financial risk on the continuance intention. Cheng et al. [11] showed that the perception of risk related to bike-sharing services can lower consumers’ behavioral intentions toward bike-sharing services. Consumers are required to provide personal information to service providers when subscribing to bike-sharing services as well as store service-related data, such as residence, frequently used routes, and usage patterns on the mobile applications [3]. In case the privacy of service providers is compromised, the stored personal information may be misused or sold to third parties. Therefore, we expect that financial and privacy risk have negative impacts on consumers’ continuance intention to use bike-sharing services.

This study aims to solve the following research questions:

(1) What are the enablers that promote customers’ intention to continue using bike-sharing services?
(2) What factors are preventing customers from continuing to use bike-sharing services?

This study contributes to the emerging literature on bike-sharing services in two major ways. First, this study investigates the formation mechanisms of consumers’ continuance intention toward bike-sharing services. Perceived value and trust in a service provider serve as major enablers to continue using bike-sharing services. This study also examines the effects of perceived usefulness, ease of use, and enjoyment on perceived values and trust. Second, this study clarifies the effect of financial risk and privacy risk on customers’ continuance intention to use bike-sharing services, considering financial risk and privacy risk as factors hindering the intention of continuous usage. It is expected that the results of this study will help understand consumer decision-making mechanisms for the continuous use of bike-sharing services and facilitate designing policies on bike-sharing services, as well as shape the marketing activities of the service providers.

Section 2 covers the theoretical background and theoretical framework. Following this, Section 3 describes the research methodology and demographic information on survey participants. The results of the structural equation modeling (SEM) are presented in Section 4. The last section discussed the theoretical and practical implications of this study. Moreover, the section addressed the study’s limitations and outlines directions for further research.

## 2. Theoretical Background and Research Model

### 2.1. Bike-Sharing Services

The main business model for bike-sharing services is a time-based payment model. Customers who want to use a bicycle must install the service provider’s mobile application and sign up for the service. A customer can search for a bicycle in the place he/she wants to use, scan the quick response code on the bicycle, or enter the bicycle number to unlock it [6,7,8]. Service providers can monitor the operation of bicycles in real-time and also obtain information on their location and condition. The main features of a shared bicycle service are as follows:

First, when paying for the use of shared bicycles, customers do not have to pay on-site fees but use mobile applications to pay based on their usage time. Mobile applications are also used in the bicycle booking process, dismantling locks, and returning them. Second, shared bicycles are equipped with a global positioning system (GPS) so that customers can easily search for nearby bicycles based on their current locations using mobile applications. In addition, consumers can use mobile applications to reserve, as well as return the bicycles to the designated areas.

Several studies on information systems and marketing have investigated the key antecedents of consumer use of bike-sharing services. Chen [12] verified that both perceived usefulness and perceived enjoyment are the key antecedents of customer’s usage intention toward bike-sharing services. Wu and Kim [16] classified service and consumer factors as those that influence the decision to continue using bike-sharing services. This study considers economic value, fragility, and trust as service factors. Fragility and reliability serve as key factors in the decision to continue using the sharing economy. Yoon et al. [17] identified accessibility, convenience, and stability as the main features of bike-sharing services and examined the effects of the main factors by incorporating the expectation disconfirmation model. Wang and Shon [8] considered perceived enjoyment, perceived ease of use, location convenience, and sustainability as the main motivations for movement, and examined the differences in the motivation of each group according to the frequency of use of shared bicycles. Ma et al. [4] showed that perceived value and trust play an important role in developing consumer’s usage intention and subjective well-being in the context of bike-sharing. Ma et al. [5] applied the modified TAM (technology acceptance model) to explain people’s usage intention toward bike-sharing service. They clarified the significant roles of perceived usefulness, perceived ease-of-use and perceived health in enhancing people’s usage intention toward bike-sharing service. Cheng et al. [11] identified the key factors that affect bike sharing services’ continuance intentions by employing extended technology continuance theory. They found the negative effects of perceived risks on consumers’ continuance intention.

In summary, previous studies on bike-sharing applied variables that are mainly considered in TAM into bike-sharing services [17,18]. Some studied have investigated the effects of inhibitors on consumers’ decisions to continue bike-sharing services. In this vein, this study explores the enablers and inhibitors of customer’s continuance intention toward bike-sharing service.

### 2.2. Research Model and Hypotheses

**Figure 1:**
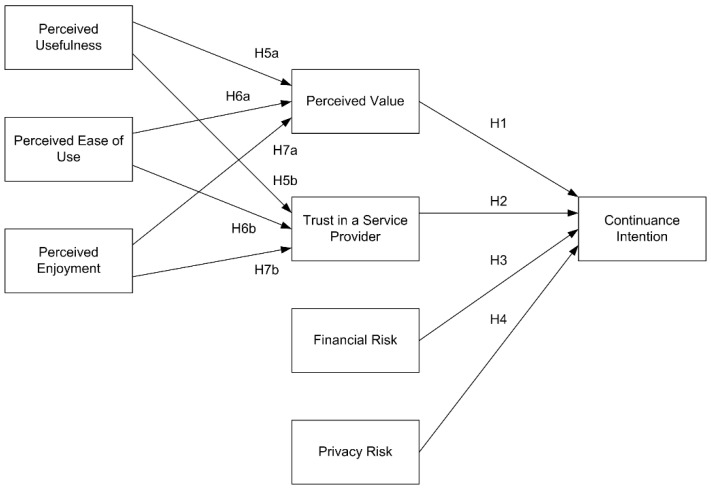
Research model.

This study examined the effects of enablers and inhibitors on the formation processes of consumer’s continuance intention in order to understand deeply their roles in consumer’s continuance behavior toward bike-sharing services. A theoretical framework of research is presented in Figure 1.

#### 2.2.1. Perceived Value

Perceived value refers to the monetary and psychological assessment based on the costs and benefits of using bike-sharing services [19]. Consumers are known to consider the benefits they derive from an action and the money and psychological costs they have to pay when taking that action. If the benefits of doing something outweigh the costs to be paid, a high level of value is perceived [20,21,22]. In other words, consumers are more likely to act in a manner that maximizes the value. In the context of bike-sharing services, consumers can also gain functionality, convenience, and pleasure from using the service although they also have to pay service fees and deposits [3]. Wu and Kim [16] verified the significant effect of perceived value on consumer decision to continue bike-sharing services. Ma et al. [4] also clarified the significance of perceived value on consumer’s usage intention toward bike-sharing services. Therefore, the more consumers believe in the worth of bike-sharing services, the more likely they are to continue using the service [20,21,22]. Therefore, it is expected that perceived value plays a key role in the formation of intent to continue using bike-sharing services.

**H1.**  *Perceived value positively influences customers’ continuance intention to use bike-sharing services.*

#### 2.2.2. Trust in a Service Provider

Trust refers to the extent of the belief that the other party will act based on positive expectations rather than opportunistic behavior [23]. According to the social relations theory, trust in a company, even in customer and business relationships, is a key factor in shaping a long-term relationship [24]. When consumers establish a reliable trust relationship, they will use the product or service more frequently and will also be willing to voluntarily make positive word-of-mouth recommendations [25]. Furthermore, trust in a service provider would play a key role in the formation of intent to sustain the use of bike-sharing services, which require revealing personal information, such as mobility as well as demographic information [3]. If the information provided by service consumers is collected and utilized illegally or without their consent, it will not only reduce trust in the service provider but also result in the eventual suspension of the use of the service. Wu and Kim [16] illustrated that trust in a service provider is related to protecting their personal information without user formal consent. They showed that trust positively influences the formation of an intention to continue using the service. Ma et al. [4] also showed that trust in a service provider plays an important role in enhancing consumer’s usage intention in the context of bike-sharing context. This study also predicts that as trust levels in bike-sharing services increase, the willingness to continue using the services would improve.

**H2.**  *Trust in a service provider positively influences customers’ continuance intention to use bike-sharing services.*

#### 2.2.3. Financial Risk

Financial risk is defined as the risk of potential financial loss that consumers perceive when using bike-sharing services [26]. Potential financial losses may arise if there is a possibility of a loss of investment when using the service, or if financial transactions such as mobile banking and deposit are conducted. Zhang and Park [27] found that financial risk inhibited the formation of satisfaction with car-sharing services. In particular, even for bike-sharing services that perform non-face-to-face services, financial risk will negatively impact consumer decisions to use the services. Financial risk in bike-sharing services may arise from the failure of a deposit refund when attempting to terminate the service [9]. In addition, a lack of bicycles and bicycle breakdown may lead to potential costs. Li et al. [15] found that financial risk in bike-sharing services was a factor inhibiting the formation of consumers’ continuance intent. This study is expected to show that financial risk harms the formation of continuance intention toward bike-sharing services.

**H3.**  *Financial risk negatively influences customers’ continuance intention to use bike-sharing services.*

#### 2.2.4. Privacy Risk

Privacy risk is defined as the extent to which it is difficult for consumers to control the conditions for collecting or utilizing information about themselves [28]. Privacy risk is considered as a key factor in consumer decision-making when purchasing goods or using non-face-to-face services, such as social network services and e-commerce. Especially for social network services, consumers may have to fill out personal information, such as gender, school, or residential area to sign up for the service [29]. Moreover, sensitive information about individuals may also be exposed during the process of posting their writings or photos. In the context of bike-sharing services, individuals also provide personal information to sign up for the service, although bike usage information about the route they followed while using the service is also stored. If the GPS of a shared bicycle is hacked, their movements and locations may be exposed. Risk factors for privacy invasion against bike-sharing services will lower the level of intent to continue using the services. Change [11] also found the negative impacts of perceived risks on consumers’ usage intention toward bike-sharing services. Therefore, this study expects that privacy risk has a negative effect on the intention to use it continuously.

**H4.**  *Privacy risk negatively influences customers’ continuance intention to use bike-sharing services.*

#### 2.2.5. Perceived Usefulness

Perceived usefulness is defined as the extent to which the act of using bike-sharing services will be achieved efficiently [13,14]. According to the IT motivational theory, perceived usefulness acts as an external motive when consumers make decisions to use particular information systems [30]. The more consumers perceive that they can use particular information systems to achieve the desired results more efficiently, the more positive they will be about the information systems. Therefore, the technology acceptance model suggests that perceived usefulness has a significant effect on consumers’ decisions to accept or continue to use information systems in various contexts [13,31]. Perceived usefulness serves as a key factor in increasing the value of the service, as bike-sharing services can be used to quickly and efficiently reach the destination in cities where vehicle mobility is inconvenient or difficult [4,15,16]. In addition, the more useful are the bike-sharing services perceived to be, the greater is the level of trust in the service provider. This is because the level of trust in a service provider increases if the consumers can achieve their goals in a useful manner through shared services rather than owning bicycles. Ma et al. [4] clarified the significant effect of perceived usefulness on trust in a service provider. Chen [12] also demonstrated the vital role of perceived usefulness in enhancing customer’s attitude and intention toward bike-sharing services. Therefore, it is expected that perceived usefulness would positively affect perceived value and trust in a service provider.

**H5a.**  *Perceived usefulness positively influences perceived value.*

**H5b.**  *Perceived usefulness positively influences trust in a service provider.*

#### 2.2.6. Perceived Ease of Use

Perceived ease of use refers to the extent to which consumers perceive bike-sharing services to be easy to use [13,14]. The easier the service use is perceived to be, the more value and confidence it generates for the user [31]. In particular, as customers of bike-sharing services use bicycles for the desired amount of time where they need, not where they own them, perceived ease of use would be a key factor in decision-making about using bike-sharing services [15,16]. Further, consumers can perform these tasks using mobile applications, without the need to come face-to-face with the service providers when unlocking or paying for bicycle use. Therefore, the more convenient is the availability of service to consumers, the higher is the perceived value of the service and the higher is the level of trust in a service provider [4,11]. Wang and Shon [8] noted that perceived ease of use is a success factor to adopt and use bike-sharing services. Ma et al. [4] found a vital role of perceived ease of use in enhancing trust in a service provider. Therefore, it is expected that perceived ease of use has a significant impact on perceived value and trust in a service provider.

**H6a.**  *Perceived ease of use positively influences perceived value.*

**H6b.**  *Perceived ease of use positively influences trust in a service provider.*

#### 2.2.7. Perceived Enjoyment

Perceived enjoyment is defined as the extent to which a user feels pleasure by using a bike-sharing service [13]. IT motivational theory states that perceived enjoyment is considered as an internal motive for using information systems and services and is used as a key clue during the consumers’ decision-making process [30]. In particular, according to the emotional processing theory, pleasant experiences of consumers are used as a key clue in determining the repurchase of the service [31,32]. Consumers’ pleasant and delightful experiences with bike-sharing services will enhance their value and confidence in those services [8,13,31]. Chen [12] verified that both perceived usefulness and perceived enjoyment are served as the key enablers to improve customer’s usage intention toward bike-sharing services. Wang and Shon [8] demonstrated that pleasant experiences in shared services played a key role in building trust, satisfaction, and value for the company. Thus, it is expected that the pleasant experience of bike-sharing services has a significant impact on improving the value of the service and increasing trust in the service provider.

**H7a.**  *Perceived enjoyment positively influences perceived value.*

**H7b.**  *Perceived enjoyment positively influences trust in a service provider.*

## 3. Research Methodology

### 3.1. Instrument Development

The survey method was used to analyze the proposed research model. To ensure the validity of the factors considered in this research model, the survey questions were drawn from existing literature, such as information systems and service management. Three researchers from related fields reviewed the survey questions, and a Chinese researcher fluent in Korean translated the questionnaire in the Korean language into Chinese and conducted a survey of Chinese bike-sharing service consumers. The translation and survey forms and expressions were modified to reflect the opinions of researchers. All survey questions were measured based on a seven-point Likert-type scale (1 = strongly disagree, 7 = strongly agree). Table A1 lists the survey items of the model constructs with related references.

### 3.2. Subjects and Data Collection

This study conducted online surveys for consumers who had used bike-sharing services in China’s first- and second-tier cities during May 2019. A total of 224 Chinese consumers using bike-sharing services were selected for data analysis. Hellobike was the most popular, with 105 (40%) people, followed by Mobike (*n* = 72) and Ofo (*n* = 47). Other demographic characteristics of the final data are presented in Table 1.

## 4. Research Results

We used PLS techniques, which offer the advantage of having fewer restrictions on the distribution of sample size and residuals compared to structural equations of covariance techniques such as LISREL and AMOS [33], to verify our model and hypotheses. The PLS technique is widely used in information systems and marketing. We performed a two-step analysis of the measurement model and the structural model for our study.

### 4.1. Measurement Model

The measurement model examined the reliability, convergent validity, and discriminant validity of the measurements. To verify the reliability, composite reliability (CR) and average variance extraction (AVE) were calculated. If the CR had a value greater than 0.70, and the AVE was greater than 0.50, the reliability was satisfied [34]. As shown in Table 2, the CR and AVE values of the factors considered in this study were satisfied, with a reliability above the threshold value. Next, convergent validity was achieved when the factor load of the survey questions was 0.70 [35]. As the factor loadings showed a value of 0.7 or higher (Table 2), convergent validity was also ensured. Finally, the discriminant validity was satisfied because the square root value of AVE for all factors was greater than the correlation value for that column or row [34]. The analysis of discriminant validity is shown in Table 3. In addition, variance inflation factors (VIFs) were calculated. VIFs of constructs indicate that multicollinearity is no serious concern.

### 4.2. Structural Model and Hypothesis Testing

**Figure 2:**
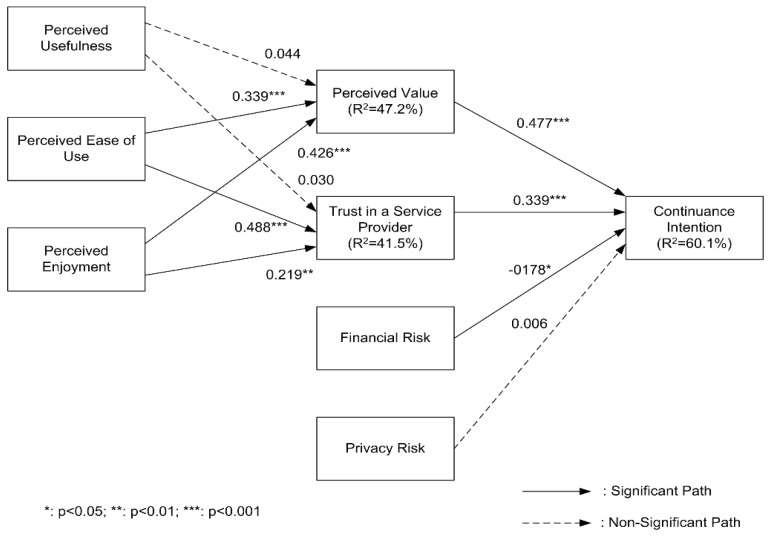
Analysis results.

An SEM was conducted to evaluate the hypothesized paths among the constructs using PLS. A bootstrap resampling method with 500 resamples was conducted to check the significance of the hypotheses within the research model. The results of the analysis are illustrated in Figure 2.

The research model was analyzed by resampling the number 500. The results of the analysis are presented in Figure 2. As expected, both perceived value and trust in a service provider had a significant impact on consumers’ continuance intention toward bike-sharing services. However, financial risk was significantly related to consumers’ continuance intention while privacy risk did not significantly affect consumers’ continuance intention. The proposed study model described 60.1% of the variance of consumer’s continuance intention. Perceived usefulness did not significantly affect perceived value and trust in a service provider. Both perceived usefulness and perceived enjoyment had a significant impact on perceived value and trust in a service provider. The proposed model accounted for 47.2% of the variance in perceived value and 41.5% of the variance in trust in a service provider. Table 4 summarizes the study’s results.

## 5. Discussion and Implications

### 5.1. Summary of Results

The research framework investigated the effects of enablers and inhibitors on customer’s continuance intention toward bike-sharing service. The findings of this study indicate that perceived value and trust in a service provider are the critical enablers of consumer’s continuance intention. These results verify that consumer’s continuance intention is shaped by perceived value as well as trust in a service provider [4,10]. From the perspective of inhibitors, financial risk is serviced as a key antecedent to hesitate to use bike-sharing services. However, in contrast to the expectations, privacy risk is not significantly related to consumer’s continuance intention. In line with our findings, Cheng et al. [11] also showed that perceived risk is not significantly associated with continuance intention in the context of bike-sharing services. In summary, customer’s continuance intention was largely explained by enablers such as perceived value and trust in a service provider, suggesting that the enablers are stronger antecedents of continuance intention than the inhibitors.

The analysis results show that perceived ease of use and perceived enjoyment were positively related to perceived value. This study also confirms the significance of trust in a service provider on the formation of consumer’s continuance intention toward bike-sharing services. Consumers with higher perceptions of ease of use and enjoyment had enhanced their levels of value and trust in a service provider, leading to an enhancement of continuance intention toward bike-sharing services [5,12,17]. However, in contrast to our expectations, perceived usefulness did not influence both perceived value and trust in a service provider. This is because a unique and authentic experience has a stronger effect on both perceived value and trust in a service provider than extrinsic motivations.

### 5.2. Theoretical and Practical Implications

This study investigated the key antecedents that affect consumers’ intention to continue using bike-sharing services. It was empirically demonstrated that perceived value plays a key role in the formation of consumers’ continuance intention. Because shared economic services can share idle resources with others and use them at low prices, perceived value plays an important role in shared economic services. The higher the consumers assign the value of bike-sharing services, the more likely they are to continue using them. Bike-sharing services, in particular, are more likely to be used for short distances or in places where it is difficult to travel by car. Therefore, it is necessary to subdivide and fine-tune consumers who have these needs. When a person travels to another city, he/she can also increase the value by providing consistent services. In addition, regular service providers need to increase the value of their services by offering regular tickets at low prices. China’s Hellobike offered a ticket for about 20 yuan (3500 won) on Thursday, designing the service so that consumers could freely use the service for a month. MotorTrend Korea also argued that COVID-19 could be an alternative to public transportation in narrow spaces. City Bike, a shared bicycle service in New York, USA, analyzed the number of consumers from March 1 to 11 and found that the usage rate increased by 66% compared to the corresponding period of the previous year [36]. For bike-sharing service providers, it is necessary to sensitize customers about the value of safety and environmental protection resulting from the use of these shared bicycle services.

Next, trust in a service provider was shown as a key factor in the formation of an intention to continue use. Due to the nature of shared services, consumers store their basic demographic information as well as information about the use of bicycle services in service providers’ mobile applications. If a bike-sharing service provider uses information about consumers’ bicycle use, such as patterns of use of bicycles and frequently used routes, for marketing purposes without user consent, or sells it to other marketing companies, then consumers will lose confidence in the service provider and eventually stop the service. Therefore, for bike-sharing service providers, it is necessary to provide consumers with transparent guidelines for storing and utilizing personal information so that consumer confidence in the service provider increases. Negative experiences of bike-sharing services lower the trust in the service provider. Hence, the service provider needs to determine the bicycle condition and the reason for its failure in real-time through GPS and other applications and rectify it quickly to prevent distrust in the service provider.

The analysis results showed that financial risk significantly affects consumers’ continuance intention toward bike-sharing services. Financial risk related to bike-sharing services is considered an important factor to form consumer’s continuance intention. Although Ofo, China’s top bike-sharing company, had a problem related to deposits paid in advance by consumers, our findings would imply that financial risk negatively affects the formation of consumers’ intent to continue using their services. This is because excessive competition weakened small bike-sharing companies that withdrew their businesses or merged with other big companies, resulting in reduced service supply. The recent issue of refund of deposits for bike-sharing services has raised doubts about the business revenue model of bike-sharing services. Lee and Kim [9] conducted text mining for Chinese shared bicycle services, which indicated that deposit problems of bike-sharing services, such as deposits and loans, were frequently mentioned on SNS. In particular, as China’s shared bicycle services became popular, by 2016, more than 100 related companies were involved in these services. Consequently, smaller companies suffered due to deposit or loan problems. However, privacy risk did not significantly affect the formation of consumers’ continuance intention. This is because service providers offer customers with guidelines on privacy and develop a variety of security technologies such as facial recognition and QR codes. In China, payments are carried out through facial recognition, which is used as a security tool during transactions. However, despite this privacy risk, our analysis revealed that consumers consider more value or trust to be gained than privacy violations while using bike-sharing services.

Perceived enjoyment had a significant impact on perceived values and trust. The main reason for the success of bike-sharing services is convenience, such as payment and unlocking bicycles through mobile applications. Major consumers of bike-sharing services belong to the 20s and 30s age-group, a generation that is very familiar with digital devices. The convenient functions offered by bike-sharing services were found to play a vital role in enhancing the value of the service and trust in a service provider. Perceived enjoyment also had a significant impact on perceived values and trust in a service provider. In the service industry, the experiences of pleasure and touch can be seen as crucial signs for continuance intention decisions. This study also empirically demonstrated that the internal motivation of bike-sharing services is a critical factor for perceived value and trust. However, perceived usefulness had no significant impact on both perceived value and trust in a service provider. This is because perceived ease of use and perceived enjoyment have a greater impact on perceived value and trust in a service provider than on the usefulness of shared bicycle services. In other words, for the bike-sharing services, it was confirmed that internal motivation and convenient functions have a greater impact on the value of the service and the trust in the provider than external motivation. In line with Kim et al. [25], it was found that the impact of internal motivation on continuous use decisions was stronger than external motivation in the context of bike-sharing services.

### 5.3. Limitations and Future Research Directions

The limitations of this study and its future direction are as follows. First, given this study only targeted Chinese bike-sharing service providers, consumer perceptions and attitudes of Chinese service providers could influence the research results. As bike-sharing services are not active in South Korea, research results from Chinese bike-sharing services can help design domestic service policy and marketing strategies in South Korea. However, for the generalization of research results, it is necessary to verify the research model for the use of bike-sharing services in South Korea. Second, in terms of financial risk, it is also considered meaningful to examine the differences in perception and attitudes between bike-sharing companies that have suffered financial difficulties, such as Ofo, and companies that have expanded through mergers and acquisitions. At a time when skepticism about the business model of the sharing economy is high, it is expected that the differences between these two groups of companies will help them understand the differences in their perceptions and develop effective strategies.

## 6. Conclusions

This study proposes a theoretical framework to explore the formation mechanisms of consumer’s continuance intention in the context of bike-sharing services. This study posits perceived value and trust in a service provider as major enablers to user’s continuance intention. Moreover, this study examines the negative effects of financial risk and privacy risk on customers’ continuance intention. The theoretical framework is conducted as a preliminary test using survey data from of 224 Chinese consumers using bike-sharing services; the data is analyzed using PLS. The analysis results clarified the significant role of perceived value and trust in a service provider in enhancing consumer’s continuance intention. However, our findings showed that financial risk negatively influences customer’s continuance intention, while privacy risk is not significantly related to it. Understanding the formation mechanisms of consumer’s continuance intention enables bike-sharing practitioners to employ more targeted marketing and service operations.

## Figures and Tables

**Table 1:** Profile of respondents.

Demographics	Item	Subjects (*N* = 224)	
		Frequency	Percentage
Gender	Male	104	46.4%
	Female	120	53.6%
Age	Less than 25	99	44.2%
	26–35	78	34.8%
	More than 35	47	21.0%
Average Income per Month	Less than 3000 yuan	84	37.5%
	3001–7000 won	95	42.4%
	7001–10,000 won	32	14.3%
	More than 10,000 won	13	5.8%

**Table 2:** Scale reliabilities.

Construct	Item	Mean	St. dev.	Factor Loading	VIF	Cronbach’s Alpha	CR	AVE
Continuance intention	COI1	5.812	0.987	0.832	1.682	0.794	0.879	0.707
	COI2	5.92	0.946	0.838	1.705			
	COI3	5.848	1.045	0.853	1.651			
Perceived value	PEV1	5.612	1.067	0.829	1.641	0.781	0.872	0.695
	PEV2	5.585	0.996	0.853	1.747			
	PEV3	5.665	1.089	0.819	1.523			
Trust in a service provider	TRU1	5.938	0.884	0.718	1.249	0.694	0.832	0.623
	TRU2	5.683	1.023	0.863	1.643			
	TRU3	5.455	1.145	0.781	1.450			
Financial risk	FIR1	5.402	1.612	0.745	2.092	0.900	0.925	0.806
	FIR2	5.415	1.59	0.957	4.741			
	FIR3	5.429	1.48	0.973	4.148			
Privacy risk	PRR1	5.513	1.282	0.789	1.899	0.835	0.895	0.741
	PRR2	5.504	1.157	0.878	1.881			
	PRR3	5.638	1.195	0.910	2.058			
Perceived usefulness	PUS1	5.942	1.027	0.831	2.079	0.824	0.891	0.733
	PUS2	5.884	1.088	0.861	2.099			
	PUS3	5.906	1.19	0.875	1.619			
Perceived ease of use	PEU1	6.004	0.989	0.750	1.332	0.700	0.833	0.624
	PEU2	5.853	1.022	0.815	1.373			
	PEU3	6.085	0.948	0.804	1.391			
Perceived enjoyment	PEN1	5.379	1.166	0.867	1.877	0.819	0.891	0.733
	PEN2	5.402	1.114	0.916	2.413			
	PEN3	5.371	1.261	0.780	1.701			

**Table 3:** Correlation matrix and discriminant assessment.

	1	2	3	4	5	6	7	8
1. Continuance intention	0.841							
2. Perceived value	0.729 **	0.834						
3. Trust in a service provider	0.691 **	0.750	0.790					
4. Financial risk	−0.060	0.080	0.122	0.898				
5. Privacy risk	0.126 **	0.193 **	0.260 *	0.643 **	0.861			
6. Perceived usefulness	0.472 **	0.373 **	0.389 **	−0.096	0.030	0.856		
7. Perceived ease of use	0.661 **	0.579 **	0.615 **	−0.044	0.114	0.606 **	0.790	
8. Perceived enjoyment	0.502 **	0.609 **	0.471 **	0.145 **	0.177 **	0.288 **	0.501 **	0.856

Note: The square root of AVE values is shown on the diagonal. *: *p* < 0.05; **: *p* < 0.01.

**Table 4:** Summary of the results.

	Cause	Effect	Coefficient	T-Value	Effect Size (f^2^)	Hypothesis
H1	Perceived value	Continuance intention	0.477	5.739	0.249	Supported
H2	Trust in a service provider	Continuance intention	0.339	4.088	0.122	Supported
H3	Financial risk	Continuance intention	−0.178	2.389	0.047	Supported
H4	Privacy risk	Continuance intention	0.060	0.863	0.005	Not Supported
H5a	Perceived usefulness	Perceived value	0.044	0.522	0.002	Not Supported
H5b	Perceived usefulness	Trust in a service provider	0.03	0.356	0.001	Not Supported
H6c	Perceived ease of use	Perceived value	0.339	3.746	0.112	Supported
H6d	Perceived ease of use	Trust in a service provider	0.488	5.313	0.210	Supported
H7a	Perceived enjoyment	Perceived value	0.426	7.977	0.258	Supported
H7b	Perceived enjoyment	Trust in a service provider	0.219	3.176	0.061	Supported

## Appendix A

**Table A1:** List of Model Constructs and Items.

Construct	Item	Mean
Continuance intention [32]	COI1	I’d like to continue to use bike-sharing services.
	COI2	I’ll continue to use the shared bike service a lot in the future.
	COI3	I’ll recommend the friends around me to use the shared bike.
Perceived value [19]	PEV1	All things considered, using bike-sharing services provides very good value.
	PEV2	Using bike-sharing services is worth my money and time.
	PEV3	It is of value for me to use bike-sharing services.
Trust in a service provider [23]	TRU1	I believe that service providers in bike-sharing services is faithful to its services.
	TRU2	I’m sure the service providers in bike-sharing services will not intentionally harm my interests.
	TRU3	I believe in the information provided by service providers in bike-sharing services.
Financial risk [28]	FIR1	I’m afraid the deposit will not be returned in time, causing property damage.
	FIR2	I’m concerned that the transaction will be illegally accessed and used during mobile payments.
	FIR3	I’ll worry about the loss of property.
Privacy risk [28]	PRR1	I’m worried that my personal information and transactional information are leaked.
	PRR2	I think that after using bike-sharing services, you’re vulnerable to junk text messages.
	PRR3	I am concerned that the service providers in bike-sharing services will collect my personal data without telling me.
Perceived usefulness [31]	PUS1	I think bike-sharing services is useful.
	PUS2	Using bike-sharing services improves travel efficiency
	PUS3	I think it’s necessary to use bike-sharing services.
Perceived ease of use [31]	PEU1	Using a shared bicycle is easy for me
	PEU2	Learning to use a shared bicycle requires less effort
	PEU3	The steps for using a shared bicycle are simple
Perceived enjoyment [31]	PEN1	Using bike-sharing services gives me a lot of fun.
	PEN2	Using bike-sharing services to make my life more interesting
	PEN3	I find using bike-sharing services interesting rather than boring
